# Organic extracts from *Cleome droserifolia* exhibit effective caspase-dependent anticancer activity

**DOI:** 10.1186/s12906-020-2858-0

**Published:** 2020-03-06

**Authors:** Neena Gopinathan Panicker, Sameera Omar Mohammed Saeed Balhamar, Shaima Akhlaq, Mohammed Mansoor Qureshi, Najeeb Ur Rehman, Ahmed Al-Harrasi, Javid Hussain, Farah Mustafa

**Affiliations:** 1 Department of Biochemistry, College of Medicine and Health Sciences, UAE University, Tawam Hospital Complex, P.O. Box 17666, Al Ain, UAE; 2 Natural and Medical Sciences Research Center, University of Nizwa, Nizwa, Sultanate of Oman; 3 Department of Biological Sciences & Chemistry, College of Arts and Sciences, University of Nizwa, Nizwa, Sultanate of Oman

**Keywords:** *Cleome droserifolia*, Medicinal plant, Anticancer potential, Apoptosis, Caspase-dependent cell death, Autophagy

## Abstract

**Background:**

This study investigated the anticancer potential of the medicinal herb, *Cleome droserifolia* (CD)*,* a local plant of the Arabian Peninsula. *C. droserifolia* is traditionally known for its rubefacient, anti-diabetic, anti-oxidant, and anti-inflammatory properties.

**Methods:**

Organic fractions of the aerial parts of *Cleome droserifolia* harvested from the Arabian Peninsula were tested in human breast and cervical cancer cell lines for their anticancer potential. This was accomplished by using biochemical and cellular assays, including MTT, caspase Glo, western blot, and annexin V/propidium iodide-based flow cytometry analyses.

**Results:**

Test of the dichloromethane fraction of the methanolic extract of *C. droserifolia,* (CDD) revealed potent cytotoxic activity (from 70 to 90%) against several human cancer cell lines, including MCF-7, MDA-MB-231, and HeLa. Further characterization of the CDD fraction in MCF-7 cells revealed that it could activate the enzymatic activity of various caspases in a statistically significant manner, and induce cleavage of both caspase 7 and poly ADB ribose polymerase (PARP) proteins, but not the ethyl acetate fraction. Test of the ability of CDD to induce early signs of apoptosis was validated by annexin V/propidium iodide assay using FACS analysis. Induction of apoptosis was completely reversed by the classic pan inhibitor of apoptosis, Z-VAD-FMK, reducing early apoptosis from 29.7 to 0.6%, confirming that CDD could induce caspase-dependent apoptosis.

**Conclusions:**

Altogether, our results reveal that *C. droserifolia* is a valuable medicinal plant with bioactive molecules that can induce apoptosis in human cancer cells. Thus, this plant should be explored further for its potential as an anticancer natural therapy as well as the isolation of novel molecules with anticancer properties.

## Background

*Cleome droserifolia* (Forssk) Del. *(syn. Roridula droserifolia Forssk.),* locally known as “*samwa*”, is an aromatic flowering shrub of the genus *Cleome* with ~ 200 species, belonging to the family Cleomaceae [1–3]. Other terminologies include *rem el bard*, *afeen*, and *mashta* in Arabic, while forssk in English. In addition, it is also known by other names such as “spider flower” and “mountain bee plant” [2–5]. All of the *Cleome* species grow at similar locations with different soil types. Moist places and rocky regions are favored for some species, while others grow in black fertile soil and rainy season, regions with waste water, and some in shaded areas in red soil which develops in warm, temperate, and moist climate during rainy season [2–5]. *Cleome* is found in tropical and subtropical countries in the Old and New Worlds, as well as in North Africa and Indian subcontinent [2–5].

*C. droserifolia* is an important species of *Cleome* due to its historical use in traditional medicine that is becoming increasingly endangered [4, 6, 7]. Plants in the *Cleome* genus improve stomach aches and treat many ailments like scabies and rheumatic fever [4–7]. They have immediate effect on abdominal and rheumatic pain, control inflammation, and are also effective towards wound healing, and snake & scorpion bites [4, 6–8]. These effects are attributed to their rubefacient, antimicrobial, analgesic, antipyretic, antioxidant, and anti-inflammatory activities [8–11]. For example, essential oils from three different species of *Cleome,* including *C. droserifolia,* were shown to have strong antibacterial properties owing to the essential oils being enriched in sulfur- and nitrogen-containing compounds [12]. *C. droserifolia* especially is well-known for its hypoglycemic effects, improving carbohydrate & lipid metabolism, fighting obesity, and enhancing antioxidant activity in diabetic rats & mice [13–20]. It also has anti-urinary schistosomiasis effects [21, 22].

*C. droserifolia* is rich in phytochemicals and several bioactive constituents have been isolated from this species (reviewed in [23, 24]). Numerous studies of *C. droserifolia* have revealed the presence of flavonoids, glycosides, carbohydrates, cardenolides, saponins, sterols, tannins, catechins, triterpenes, and sesquiterpenes, such as buchariol, teucladiol, daucosterol, and a new alkaloid from the aerial parts [22–30]. Other than these compounds, it has the distinction of being the first plant source of diterpenoid dolabellane esters as well [31]. Other *Cleome* species have been shown to contain numerous flavonoids glycosides [32–34], kaempferol 3-glucuronide from roots [35], a new naringenin glycoside [36], three new coumarino lignoids from seeds [37], and others [23, 24, 38]. Some of these constituents are thought to be responsible for the hypoglycemic effect of *Cleome* in animals [17–19] as well as its liver-protective properties [17, 39]. Thus, the isolation of several new phytonutrients from *Cleome* makes it an attractive candidate for further drug discovery [23, 24, 40].

Not much is known about the anticancer potential of *Cleome. Cleome gynandra,* has been shown to be effective when injected in Swiss albino mice using Ehlrich’s ascites carcinoma cells [41]. Similarly, extracts from another species, *Cleome arabica,* has been shown to have cytotoxic effects against the mouse leukemia cell line P388 by activating apoptosis and inhibiting phosphorylation of AKT and ERK kinases induced by the epidermal growth factor signaling [42]. Some of these cytotoxic effects could be attributed to the presence of dammarane triterpenes in these extracts that have been shown to have cytotoxic effects in P388 cells in MTT assays [28].

The aim of this study was to investigate the cytotoxic potential of *C. droserifolia* in more detail and determine its mechanism of action as part of the collaborative work being undertaken to collect, categorize, and study the biological potential of native land and marine flora and fauna of the Sultanate of Oman. Towards this end, the native *C. droserifolia* species was collected and identified and the aerial parts of the plant were used to prepare various organic fractions from its methanolic extract [30]. This was followed by test of the anticancer potential of these samples on breast and cervical cancer cell lines. Our results confirm the cytotoxic potential of *C. droserifolia* observed in the Egyptian variety [43] and further reveal that the active anticancer agents in this species have the ability to induce potent caspase-dependent apoptotic activity in human cancer cells. This finding supports the contention that medicinal plants serve as an important and rapidly dwindling source of novel and natural biomolecules for the fight against cancer and other human ailments [7, 44].

## Methods

### Plant collection, extraction, and fractionation

**Fig. 1:**
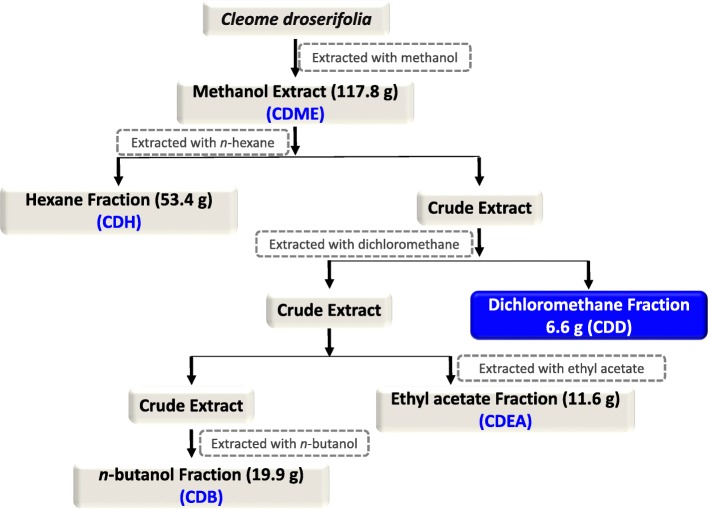
Preparation of organic fractions of *Cleome droserifolia* methanolic extract. Flow chart representing the steps used to prepare the various organic fractions of *Cleome droserifolia* (CD) starting from its methanolic extract (CDME). The residual aqueous fraction (20 g) is not shown in this scheme

The fresh whole plant material was collected from *Al-Jabel Al-Akhdar, Al-Dakhiliya* region in Oman (2012) according to local and national rules and regulations. The specie was identified by a trained taxonomist at the Department of Biological Sciences and Chemistry, University of Nizwa, Oman. The voucher specimen (JCD/02/2012) was kept in the herbarium of Natural and Medical Sciences Research Center, University of Nizwa, Oman. As described earlier [30], 7.5 kg of whole aerial parts of *C. droserifolia* were used for extraction. These parts were dried in the dark, chopped and ground to a coarse powder, followed by organic extraction with methanol for 2–3 weeks at room temperature. After evaporation under pressure (leaving a greenish syrupy residue behind), the methanol extract (CDME) was suspended in distilled water and fractionated into *n-*hexane (CDH), dichloromethane (CDD), ethyl acetate (CDEA) and *n*-butanol (CDB) fractions (Fig. 1).

To test the anticancer potential of the various *Cleome droserifolia* extracts/fractions, they were solubilized individually in the solvent dimethyl sulfoxide (DMSO) to prepare stock solutions. The concentration of the stock solutions varied for different extracts/fractions depending upon their form and solubility (12.5 mg/ml, 25 mg/ml, and 50 mg/ml), and then stored at -20 °C. From these stock solutions, different extract/fraction treatments were prepared for the MTT assays at twice the final concentration (100, 250, and 500 mg/mL) in media, depending upon the cell line used, since 100 μL of treatment was added to cells plated in 100 μL media on the day of treatment. Each experiment had control samples that contained the same concentration of DMSO as present in the tested fractions or their dilutions as a control for cell death induced by DMSO alone.

### Cells and cell culture

The hormone responsive human breast cancer cell line, MCF-7 and the triple-negative human breast cancer cell line, MDA-MB-231, were obtained from Prof. Samir Attoub, Department of Pharmacology, College of Medicine and Health Sciences (CMHS), UAE University (UAEU), Al Ain, UAE. The normal breast epithelial cell line, MCF-10A, was obtained from ATCC (USA). The human cervical cancer cell line, HeLa, was a gift of Prof. Tahir A. Rizvi, Department of Microbiology and Immunology, CMHS, UAEU. The cell lines were cultured in the ATCC-recommended media as per instructions. All cells were maintained at 37 °C in a 5% CO_2_ incubator. Cells were plated a day before conducting the experiment and seeded at the cell density mentioned in the experiments, depending upon the plate and well size used for the experiment. This was followed by treatment of the cell cultures with different concentrations of the tested extracts along with appropriate dilutions of DMSO. Passage numbers of cell lines used for different experiments can be provided to the readers upon request.

### MTT cell viability assay

All MTT [3-(4, 5-dimethylthiazol-2-yl)-2–5-diphenyltetrazolium bromide] assays were conducted in 96-well plates at a density of 5 × 10^3^ cells/well/100 μL media. Appropriate normal or cancerous cell lines mentioned above were treated with 100 μL of the different concentrations of the organic extract/fraction of *C. droserifolia*, or DMSO (the solvent), or culture media alone, for the indicated time points. This was followed by addition of 25 μl/well of the MTT stock solution (5 mg/ml) and incubation for 3–4 h at 37 °C. DMSO was used to dissolve the formazan crystals that were formed (200 μl per well), followed by measurement of absorbance at 560 nm using the Perkin Elmer Victor TM 2030 Multi-label Reader. Cell viability was calculated as a percentage of cells treated with the test extract/fraction with cells treated with DMSO alone as a control using the same concentrations of DMSO as the test treated cells. Results were analyzed using MS Excel and GraphPad Prism v5 software.

### Cell titer-GLO luminescent cell viability assay and Caspase-GLO 3/7, 8 and 9 assays

MCF-7 cells were cultured in opaque, white, 96-well plates (5000 cells/well) and treated with 50 μl of the different extract concentrations at 37 °C. The Cell Titer-Glo Luminescent Cell Viability Assay (Promega, USA) and Caspase-Glo 3/7,8 and 9 assays were used after 48 h to monitor cell viability and caspase enzyme activities, as per manufacturer’s instructions. The Infinite M200 Pro Tecan plate reader was used to measure luminescence and the resulting data was plotted as percent cell viability of experimental groups compared to DMSO-treated cells, the viability of which was taken as 100%.

### Protein extraction and Western blot analysis

Total cell proteins were extracted from the treated cells and subjected to western blot analyses for investigation of cellular pathways activated upon extract/fraction treatment. Briefly, cells treated with various concentrations of the fractions were harvested by trypsinization, washed with 1 x PBS, and lysed using 100 μL of RIPA (radioimmunoprecipitation assay) lysis buffer (10 mM Tris-Cl (pH 8.0), 1 mM EDTA, 1% Triton X-100, 0.1% sodium deoxycholate, 0.1% SDS, 140 mM NaCl) per million cells supplemented with 50 μl of β-mercaptoethanol/ml RIPA and 1 mM of the serine protease inhibitor, phenylmethylsulfonyl fluoride (PMSF). Nuclei were separated from the lysed cells by spinning at 14,000 rpm for 10 min at 4 °C. The cell lysates were separated from the pellets and either used immediately or stored at -80 °C. Protein amounts in the cellular extracts were quantified using the Bio-Rad Bradford colorimetric assay.

Total protein lysates (40 μg per lane) were loaded onto 8–12% SDS-polyacrylamide mini gels (Bio-Rad) or 4–12% gradient gels (GeneScript, USA) and blotted onto Protran nitrocellulose membranes using standard protocol. After wet transfer, the membranes were blocked with 5% low fat dried milk in 1 x PBS and 0.1% Tween-20 (PBST) for 1 h and incubated at 4 °C overnight with the specified primary antibodies in 1% milk in PBST. All antibodies were purchased from Cell Signaling Technologies (USA). After washing, the blots were incubated with the appropriate secondary antibodies (anti-mouse or anti-rabbit) for 1 h and 30 min at room temperature followed by detection using the Pierce™ ECL Plus Western Blotting Substrate and visualized using Typhoon reader.

### Annexin V/Propidium iodide flow cytometry analysis

Flow cytometry was used to test the ability of the effective extracts/fractions to induce apoptosis by inducing inversion of the phosphatidylserine phospholipid present at the inner plasma membrane to the outer layer of the phospholipid bilayer. This was accomplished by staining the treated and untreated cells with fluorescently labelled Annexin V (AV) and the vital dye, propidium iodide (PI) using a commercially available kit from Becton Dickenson, (Franklin Lakes, NJ USA). Ten thousand cells were counted per sample for flow cytometry using BD FACS Canto II, USA machine by counting all cells without gating to prevent sample bias. The apoptosis inhibitor, Z-VAD-FMK (Promega, USA), was used at a concentration 20 μM for 2 h prior to extract/fraction treatment. Z-VAD-FMK is a well-known pan-caspase inhibitor that is permeable to the cells. It binds to the catalytic site of caspase enzymes and inhibit their function, thereby preventing apoptosis.

### Morphological studies

Control and treated cell cultures were analyzed for morphological changes by observation via an inverted light microscopic attached to a CCD camera or EVOS Cell Imaging System (Thermo-Fisher Scientific, USA). Images were saved at various magnifications at different times after treatment.

### Statistical analysis

Statistical analysis was conducted using either Microsoft Excel or GraphPad Prism v5 software. In case of replicates, average values were calculated for each replicate followed by standard deviation calculations plotted as error bars on line or column graphs. Two-tailed, paired, *t*test was used to calculate significant differences between groups, while extent of significance was depicted as stars, with * = *p* < 0.05, ** = *p* < 0.01 but > 0.001, and *** = *p* < 0.001. The MTT line graphs were generated by normalizing the values obtained for the test groups with that of their DMSO control by using the DMSO control data for each concentration tested. Same was the case for the Promega Glo assays where the effect of DMSO control was used to normalize the data obtained from the treated samples. Finally, the IC_50_ values for CDD were calculated by using the non-linear regression function of GraphPad Prism v7 and least square fit to estimate the final values.

## Results

### Screening of extracts/fractions for anticancer activity

Four organic fractions from the *C. droserifolia* (CD) methanolic extract (*n*-hexane, dichloromethane, ethyl acetate, and *n*-butanol) [30] were tested for their cytotoxic potential. The solvents were used with increasing polarity to ensure that bioactive molecules of a wide range of polarity were successfully extracted from the crude plant material [44]. Interestingly, the amount of *n*-hexane fraction obtained was ~ 45% of the total extract (53.4 g out of 117.8 g total extract; Fig. 1). This may be due to the nature of the compounds present in this species that contain high concentration of monoterpenoids, diterpenoids, cembrenes, and simple triterpenoids [23, 24, 45, 46]. We have fractionated another species of the same genus (*Cleome austroarabica*) and obtained more non-polar (*n*-hexane and chloroform) than polar compounds [47].

To determine the cytotoxic potential of these extracts/fractions, an initial screening was performed using the MTT assay in the breast cancer cell line, MCF-7 [48], and the cervical cancer cell line, HeLa [49] to determine which fraction had the potential to affect proliferation of either of these cancer cell types. The four fractions were tested in a dose- and time-dependent manner at different concentrations (50, 125, 250 μg/mL) and at two different time points (24 and 72 h) post treatment. Table 1 summarizes the results obtained. As can be seen, only the dichloromethane fraction (CDD) was able to induce significant cytotoxic effects in both MCF-7 and HeLa cultures. None of the other fractions (CDH, CDEA, or CDB) were able to induce > 20% death in either of the two cell lines.

**Table 1:** Summary of initial screening of *C. droserifolia (*CD*)* organic fractions using the MTT assay

Fraction Name	MCF-7 Breast Cancer Cells*	HeLa Cervical Cancer Cells*
*n-*Hexane (CDH)	X	X
Dichloromethane (CDD)	24HR: 125/250 μg/mL 72HR: 50 μg/mL	24HR: 250 μg/mL 72HR: 125/250 μg/mL
Ethyl acetate (CDEA)	X	X
*n*-Butanol (CDB)	X	X

X = Fraction not effective (< 20% cell death) for inhibiting proliferation of either cell line

* = Concentration in microgram/ml (μg/ml) at which > or = 20% inhibition of proliferation was observed

### CDD is efficient at inducing cytotoxic effects in both Normal and Cancer cells

**Fig. 2:**
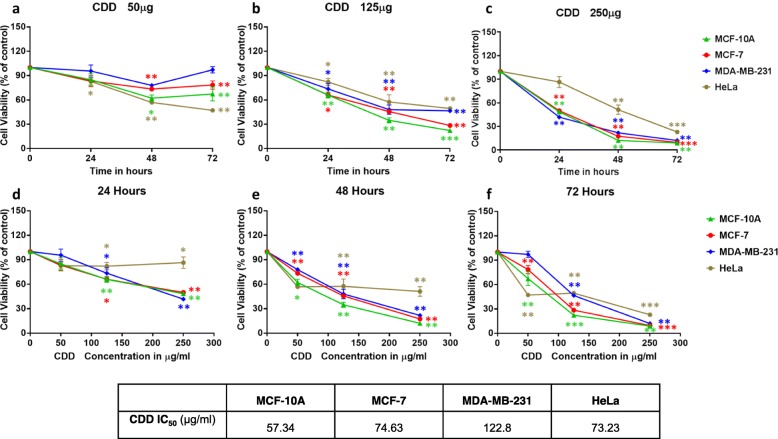
The dichloromethane fraction of *Cleome droserifolia* methanolic extract (CDD) can induce potent cell death in normal and cancer human cell lines. Cytotoxic effect of A) 50 μg/ml, B) 125 μg/ml, and 250 μg/ml of CDD using the MTT assay on MCF-10A, MCF-7, MDA-MB-231, and HeLa cells in a dose- (**a**-**c**) and time- (**d**-**f**) dependent manner, after normalizing to the effects of DMSO solvent used to dissolve the extract. * indicates statistically significant differences between the control and treated samples (* = *p* < 0.05; ** *p* < 0.01 but > 0.001; *** = *p* < 0.001). The table below the figure shows the IC_50_ values calculated for CDD in the four cell lines tested

To study cytotoxic potential of the CDD fraction further, it was tested in an expanded series of human cells, including MCF-10A, a normal breast cell line [50] and MDA-MB-231, the hard-to-treat, prototypic, triple-receptor-negative breast cancer cell line [51] along with the hormone-inducible MCF-7 and HeLa cells. First, the MTT assay was repeated in these cell lines in a dose- and time-dependent manner 24, 48, and 72 h post treatment (Fig. 2). This allowed us to compare effects of CDD in both normal and cancer cell lines at the same time and in the same manner.

Figure 2 shows results of the test of CDD on the tested cell lines after normalizing the effects of DMSO on cell proliferation, an organic solvent that was used to dissolve CDD for testing purposes. As can be seen, statistically significant cell death could be observed in three of the four cell lines, even at exposure with 50 μg/mL CDD by 48 h post treatment, except for the triple-negative cell line MDA-MB-231 (Fig. 2a). All four cell lines became susceptible to CDD-induced cell death by treatment with 125 μg/mL of CDD at 24 h, leading to death of ~ 70% of MCF-10A and MCF-7 cells and ~ 60% of MDA-MB-231 and HeLa cells by 72 h post treatment (Fig. 2b). Treatment with 250 μg/mL CDD led to induction of > 90% cell death in all the three breast cell lines tested in a comparable fashion, with HeLa cells nearly catching up by 72 h post treatment (Fig. 2c).

Time-dependent analysis of CDD-induced cell death revealed that once again, the three breast cell lines were similar in their susceptibility to CDD early on with the HeLa cells nearly catching up by 72 h post treatment with the different doses of CDD (Fig. 2d-f). Interestingly, HeLa cells were consistently more susceptible to CDD-induced killing at lower doses (50 μg/mL) than the breast cells, as observed by the steeper slope of the kill curve at 48 and 72 h (Fig. 2e & f). These subtleties were reflective in the IC_50_ values calculated at the 250 μg/ml value 72 h post treatment which revealed that the normal MCF-10A were the most sensitive to CDD-induced killing with an IC_50_ value of 57.34 μg/ml, followed by HeLa and MCF-7 cells at 73.23 and 74.63 μg/mL, respectively, while MDA-MB-231 were less sensitive with an IC_50_ of 122.8 μg/ml. These values are in the same range as observed for the cell killing potential of two other medicinal plants that we have tested in these cell lines [52, 53]. To ensure that the cytotoxicity being observed was not due to any residual organic solvent in the tested fractions, each of the organic solvents by itself was tested on the three cancer cell lines and observed to be non-toxic (data not shown). Further NMR analysis of the organic fractions also confirmed absence of residual organic solvent, confirming our results that bioactive components within *C. droserifolia* are the ones that affect cell proliferation of both human breast and cervical cancer cells significantly, but at the same time, they also affect the viability of normal cells. Finally, the fact that none of the other three fractions showed any significant signs of cytotoxicity also confirmed that residual solvents in the fractions were not responsible for the cytotoxicity being observed.

### CDD can induce enzymatic activity and cleavage of key apoptotic proteins

**Fig. 3:**
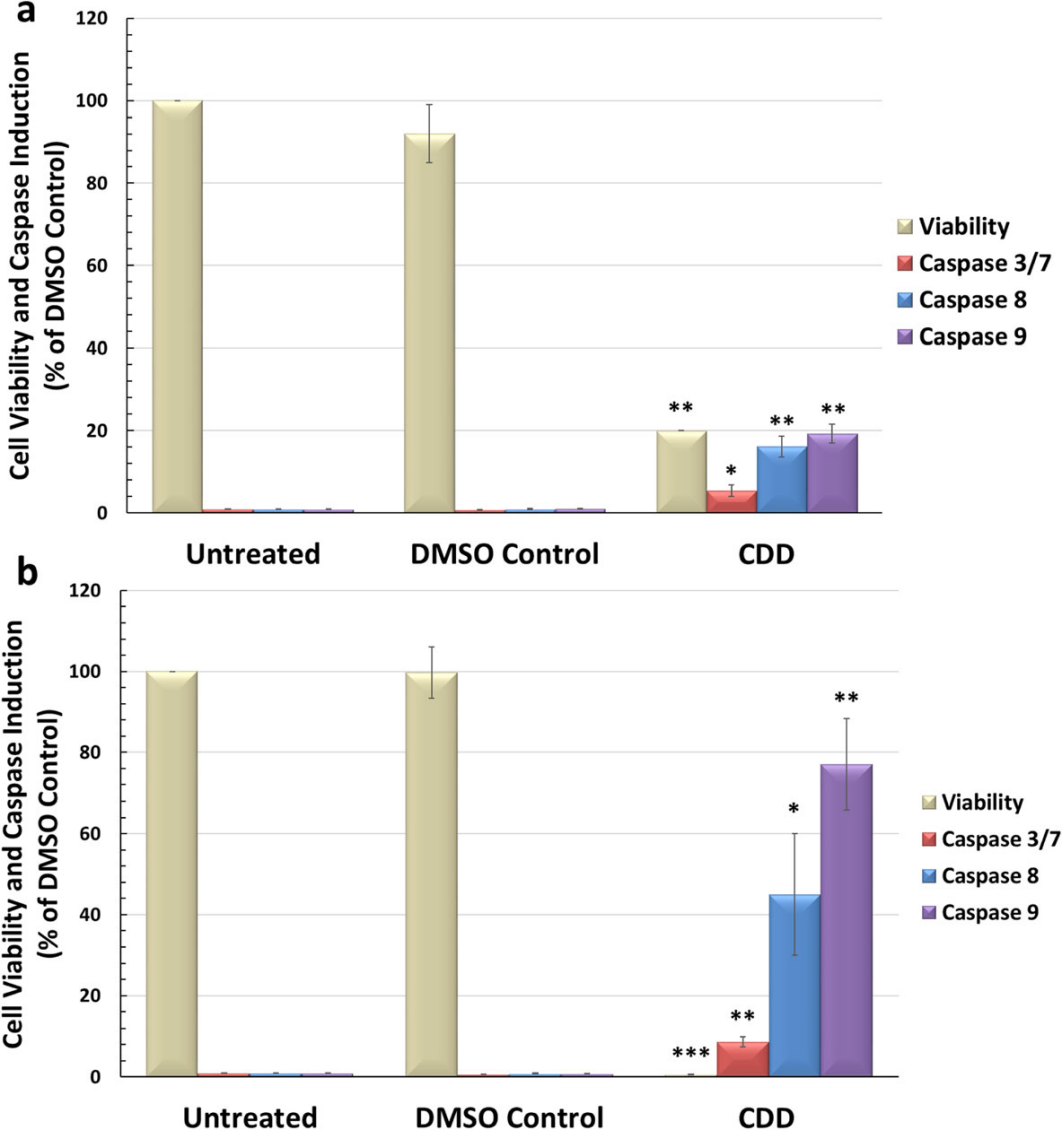
The dichloromethane fraction of *Cleome droserifolia* methanolic extract (CDD) can activate the enzymatic activity of caspase 3/7, 8, and 9. MCF-7 cells were treated with 250 μg/ml of CDD for 48 h. **a**) and **b**) show results from two independent experiments. *, statistically significant differences between the control and treated samples (* = p < 0.05; ** p < 0.01 but > 0.001; *** = p < 0.001). DMSO Control, MCF-7 cells treated with media containing the same amount of DMSO as the CDD-treated samples

To explore the potential mechanism of action of the effective extracts, the ability of CDD to induce apoptosis was explored since apoptosis is the most widely induced type of cell death pathway observed by natural compounds [54]. MCF-7 cells were treated with CDD for 48 h and assayed for its ability to induce the activity of various caspase enzymes, a hallmark of apoptosis induction [55]. As can be seen, CDD induced potent anti-proliferative effects in MCF-7 cells (> 80%) and was also able to induce the caspase enzyme activity in a statistically significant manner (Fig. 3). This was reproducible, as observed in two independent experiments conducted in triplicates (Fig. 3a and b). The levels of caspase 8 & 9 induced were higher than that of caspase 3/7. The effect on cell viability correlated well with the results of the MTT assay (Fig. 2). All results obtained for the induction of caspases were normalized to the number of viable cells in culture to ensure that the results were not skewed due to the ensuing cell death (see Methods). Since MCF-7 cells do not express caspase 3 [56], any induction of caspase 3/7 in this system is most likely due to the induction of caspase 7.

To confirm whether CDD tested in the Promega Glo enzyme assays for caspase activation could indeed induce apoptosis, our next aim was to confirm the activation of caspases associated with apoptosis using western blot analyses. MCF-7 cells were treated with CDD for 24 h and tested for the expression and cleavage of various caspases and poly ADP-ribose polymerase (PARP). PARP is one of the enzymes that is cleaved during the execution phase of apoptosis by activated caspases [55, 57]. Thus, activation of caspases and PARP by cleavage are two major indicators of a cell undergoing apoptosis.

**Fig. 4:**
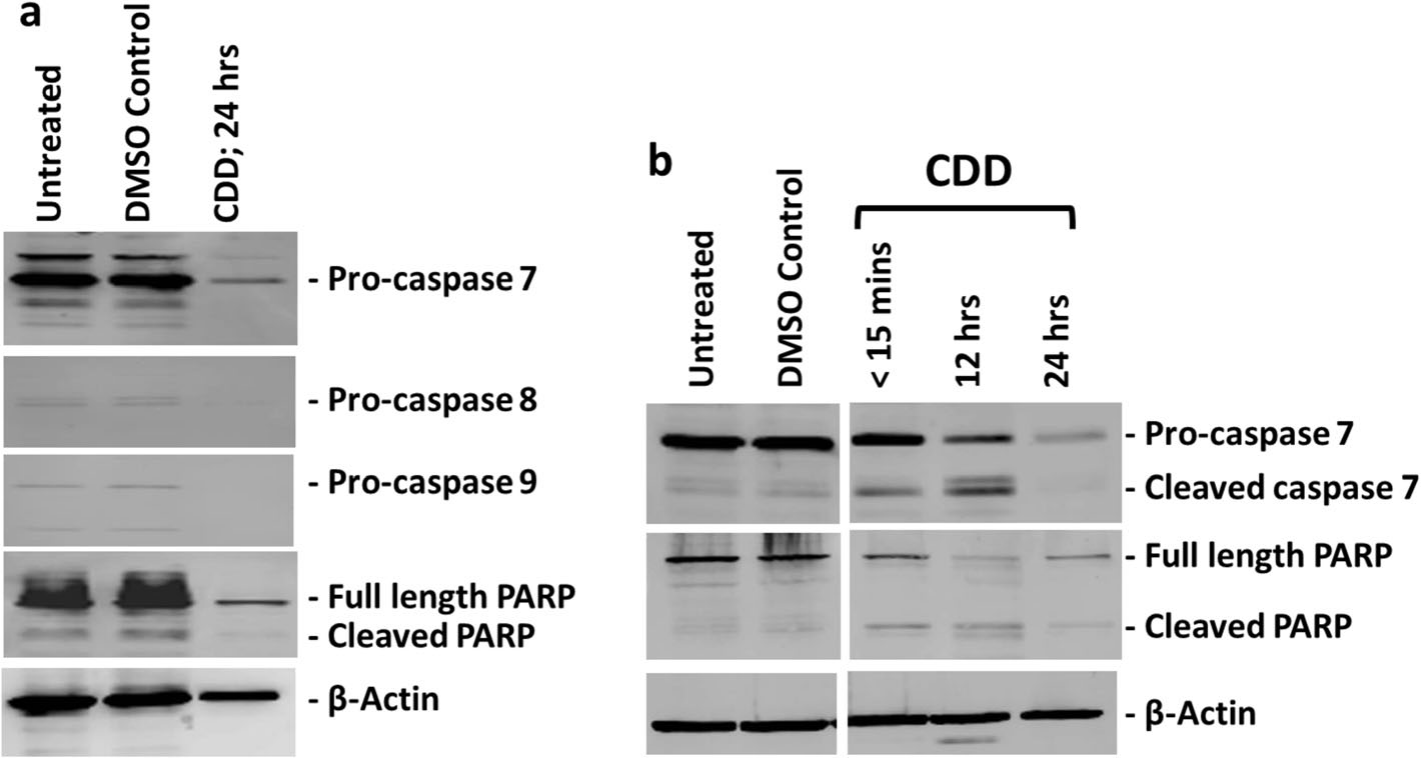
The dichloromethane fraction of *Cleome droserifolia* methanolic extract (CDD) can induce cleavage of procaspase 7 and PARP. MCF-7 cells were treated with 250 μg/ml of CDD for **a**) 24 h, and **b**) multiple time points. Western blots were carried out on the untreated, DMSO Control, and CDD-treated cellular lysates for the specified caspase or poly ADB ribose polymerase (PARP) proteins. Beta-actin antibody was used as a loading control. DMSO-treated, DMSO control having the same amount of DMSO as the test samples

Figure 4a shows the western blot analysis of CDD on MCF-7 cells 24 h post treatment. As can be seen, we could not detect a cleaved band for any of the caspase proteins and in fact, the antibody reactivity for caspase 8 and 9 was very poor for these proteins from MCF-7 cells; however, diminished amounts of procaspases 7, 8, and 9 could be observed at 24 h, suggesting that the cleavage reaction had taken place (Fig. 4a). Test of the PARP protein revealed significant reduction of full-length PARP, also suggesting its activation (Fig. 4a). To verify the results, MCF-7 cells were once again treated with the extract, but for shorter time periods in case the cleavage event had been missed due to the timing of the analysis. Figure 4b shows the results for MCF-7 cells treated for < 15 mins, 12 h, and 24 h with CDD, representing an early, intermediate, and late time point for detecting caspase cleavage. Clear cleavage of caspase 7 and PARP could be observed starting from around 15 min post treatment. The cleaved product decreased or essentially disappeared by 24 h, as observed earlier (Fig. 4b vs 4a). This observation confirms that CDD has the potential to induce apoptosis in MCF-7 cells.

**Fig. 5:**
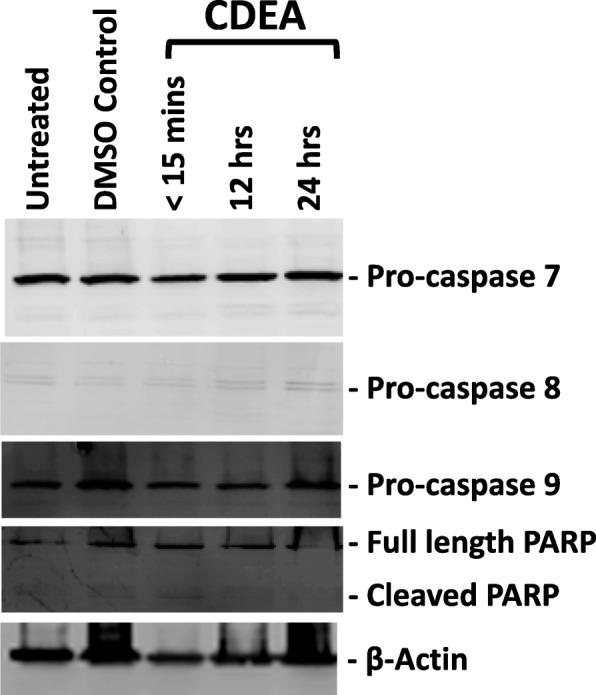
The ethyl acetate fraction of *Cleome droserifolia* methanolic extract (CDEA) is unable to induce apoptosis. Western blot analysis of MCF-7 cells treated with 250 μg/ml of CDEA for various time points and analyzed with the specified antibodies. Beta-actin antibody was used as a loading control. DMSO Control, control sample having the same amount of DMSO as the test samples

To ensure that the results being observed were truly due to the ability of CDD to cause apoptosis, we took the ethyl acetate fraction of the CD methanol extract (CDEA) that had shown an inability to cause cell death in MCF-7 cells (Table 1) and treated the MCF-7 cells with it in a similar manner (Fig. 5). Results of the western blot analysis of CDEA-treated cells revealed a complete lack of cleavage of either the procaspases 7, 8, or 9, or PARP, or decrease in their expression, confirming that the cleavage of the procaspases and PARP was truly due to the ability of CDD to induce apoptosis.

### CDD can induce early signs of apoptosis

To confirm whether CDD could indeed induce apoptosis, we tested the potential of CDD to induce the inversion of the plasma membrane phospholipids, an early sign of apoptosis [55]. Towards this end, MCF-7 cells were treated with CDD and tested for the inversion of the phosphoserine phospholipid that is found exclusively at the inner leaflet of the plasma membrane using FITC-labelled Annexin V (AV), a carbohydrate that has high affinity for phosphoserines moieties [55]. Furthermore, to determine whether the cell membrane integrity was maintained, cells were stained with propidium iodide (PI), a vital dye that can differentiate between live and dead cells. PI can enter the cells once the cell membrane integrity has been compromised in dead cells and therefore is excluded from live cells [55].

**Fig. 6:**
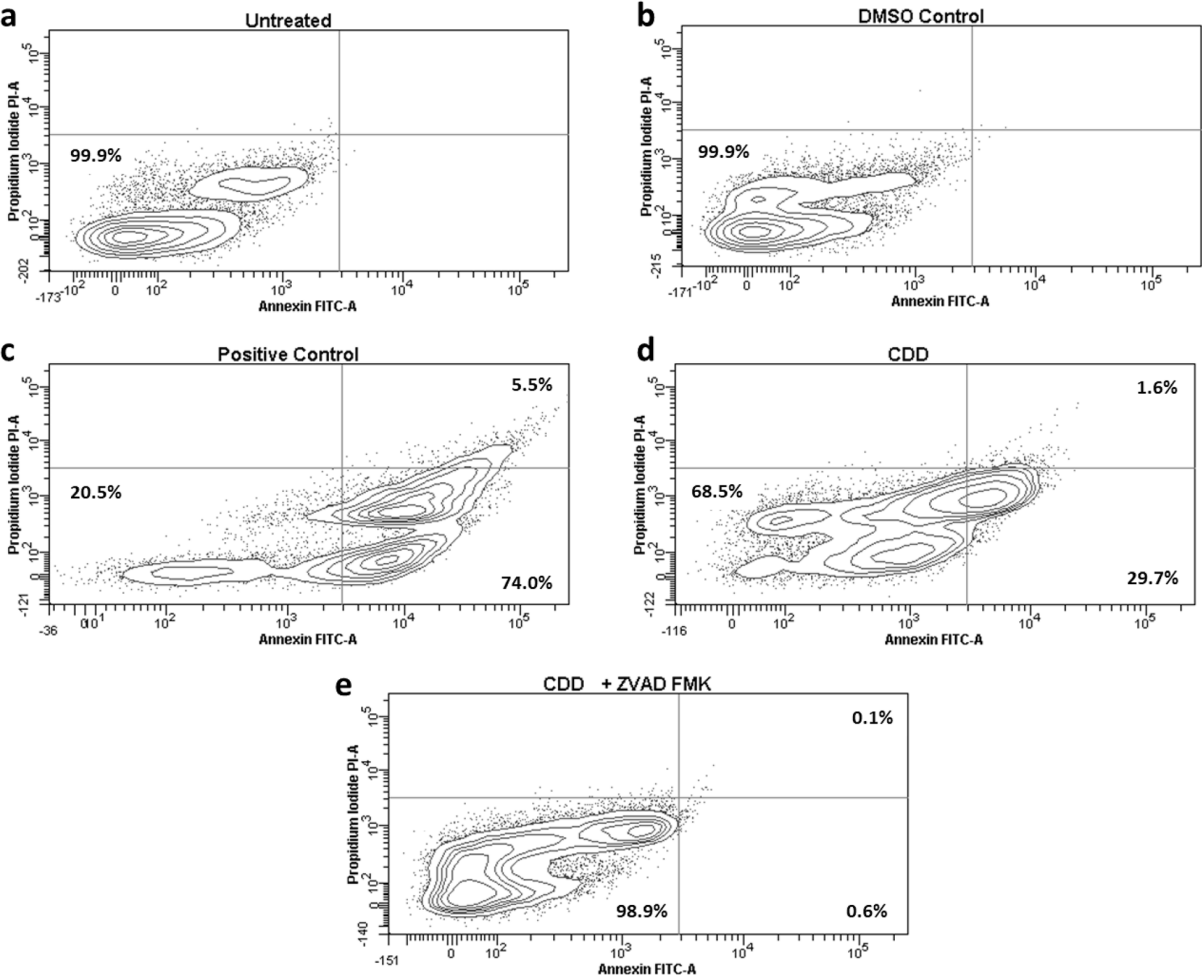
The dichloromethane fraction of *Cleome droserifolia* methanolic extract (CDD) induces inversion of inner plasma membrane phospholipids. FACS analysis of MCF-7 cells stained with Annexin V/propidium iodide after treatment with: **a** media alone, **b** media with DMSO, **c***Boswellia sacra* essential oil, a known apoptosis inducer, **d** 250 μg/ml CDD for 24 h, or E) 250 μg/ml CDD after pre-treatment with 20 μM Z-VAD-FMK for 2 h. DMSO Control, MCF-7 cells treated with the same amount of DMSO as the test samples

Figure 6 shows the flow cytometric analysis of the MCF-7 cells treated with CDD 24 h post treatment. As can be seen, 99.9% of the untreated and DMSO-treated cells were observed to be in the AV(−)/PI(−) quadrant, indicative of live healthy cells (Fig. 6a and b). Treatment with an essential oil from *Boswellia sacra* (positive control) that induces potent apoptosis in breast cancer cells [52, 53, 58] shifted 74% of the cells to the AV(+)/PI(−) quadrant indicative of early apoptosis, and 5.5% of the cells to the AV(+)/PI(+) quadrant, indicative of late apoptosis (Fig. 6c). In comparison, 29.7% of the cells were shifted to the early apoptosis by CDD and 1.6% of the cells to the late apoptosis stage (Fig. 6d). Pre-treatment of the MCF-7 cells with Z-VAD-FMK, a classical inhibitor of apoptosis [55], was able to completely reverse the cell death being observed in the CDD-treated cultures with 98.9% of the cells appearing in the quadrant indicting healthy and live cells (Fig. 6e). These results confirm that CDD has the potential to induce apoptosis in MCF-7 cells.

**Fig. 7:**
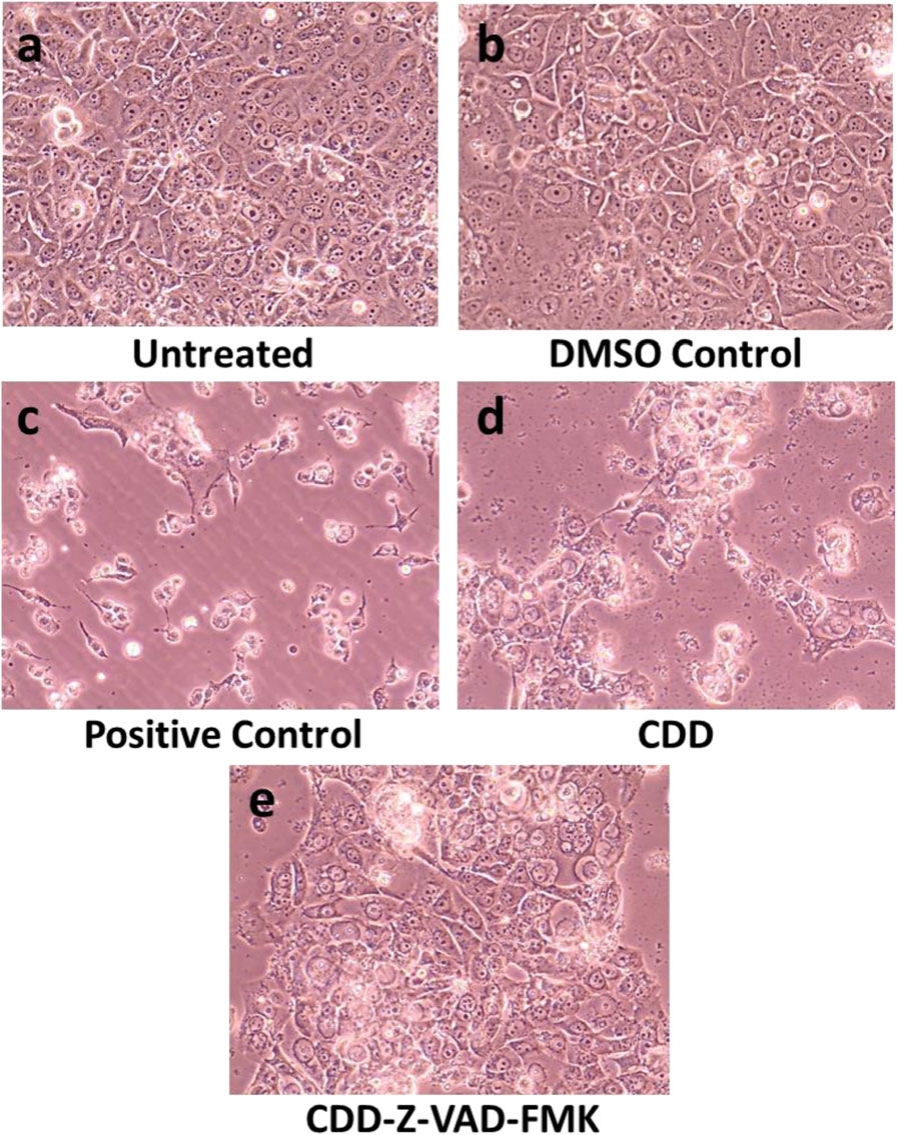
The dichloromethane fraction of *Cleome droserifolia* methanolic extract (CDD) induces morphological changes reminiscent of apoptosis in breast cancer cells. Photomicrographs of MCF-7 cells treated with **a**) media alone, **b**) media with DMSO, **c**) *Boswellia sacra* essential oil, a known apoptosis inducer, **d**) 250 μg/ml of CDD for 24 h, or **e**) 250 μg/ml CDD after pre-treatment with 20 μM Z-VAD-FMK for 2 h. DMSO Control, MCF-7 cells treated with the same amount of DMSO as the test samples. Magnification: 400X

Microscopic analysis of cells treated with CDD for 24 h revealed that unlike the untreated and DMSO-treated cells that were found to create nice monolayers (Fig. 7a and b), the CDD-treated cells exhibited cell rounding and shrinkage, characteristic of apoptosis, as observed with *Boswellia sacra* (Fig. 7c and d), followed by their removal from the monolayer. Pre-treatment of cells with Z-VAD-FMK essentially maintained the healthy phenotype of the CDD-treated monolayer (Fig. 7e), confirming that indeed CDD induces apoptosis in MCF-7 cells.

### Effect of *C. droserifolia* on autophagy

Finally, we explored the ability of CDD to activate autophagy, a type II cell death pathway normally activated by nutrient deprivation, growth factor depletion, or hypoxia [59]. Induction of autophagy is actively being explored as an important cellular pathway activated by anticancer compounds [60–63]. Our earlier work with two other medicinal plants, *Teucrium mascatense* and *Acridocarpus orientalis* has shown that induction of apoptosis by natural products can activate/perturb autophagy as well [52, 53]. Therefore, we tested the ability of CDD to induce key proteins of the autophagy pathway, the autophagy receptor, SQSTM1/p62, responsible for recognizing the ubiquitinated cellular cargo targeted for degradation, and the microtubule-associated protein 1 light chain 3 expressed in the cytoplasm (LC3-I) or its cleaved form (LC3-II) that is recruited to the autophagosome via lipidation upon autophagy activation [59].

**Fig. 8:**
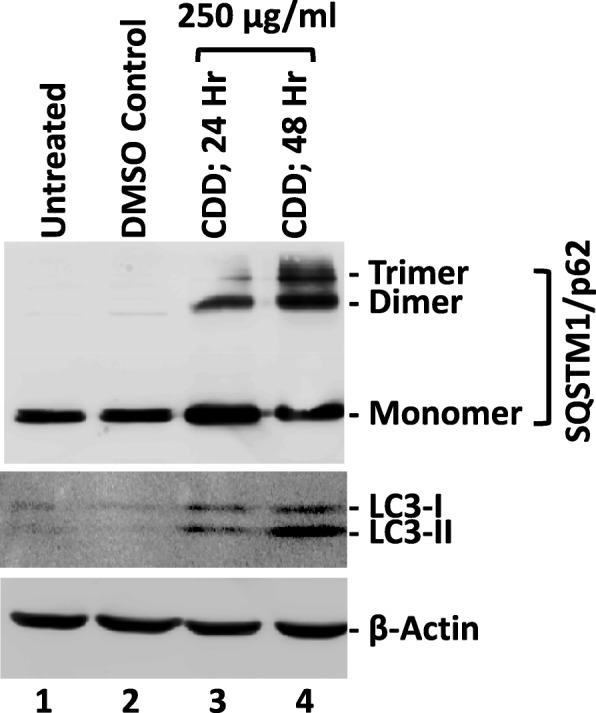
The dichloromethane fraction of *Cleome droserifolia* methanolic extract (CDD) induces expression of key proteins of autophagy in breast cancer cells. Western blot analysis of MCF-7 cells treated with 250 μg/ml of CDD for indicated time points. Beta-actin antibody was used as a loading control. DMSO-treated, DMSO control having the same amount of DMSO as the test samples

Treatment of MCF-7 breast cancer cells with 250 μg/mL of CDD led to the oligomerization of SQSTM/p62 protein (mostly dimer and some trimer formation) 24 h post treatment; the monomer, though remained the most abundant species (Fig. 8; lane 3, upper panel). By 48 h, both the dimer and trimer had increased, with the dimer being still more abundant than the trimer form, while the monomer amount decreased compared to the 24-h time point (Fig. 8; lane 4 vs lane 3; upper panel). Treatment with CDD also induced the expression of cytosolic LC3-I and the membrane-bound LC3-II by 24 h in equal proportions (Fig. 8; lane 2 vs lane 3; middle panel). This suggested recruitment of soluble LC3–1 to the developing autophagosomes, a process that continued for 48 h, as observed by the increase in LC3-II band compared to LC3-I, signifying continued activation of autophagy (Fig. 8, lanes 3 and 4; middle panel). Beta-actin served as the control with no effect of CDD either on its expression or stability Fig. 8, lanes 1–4; lower panel), confirming that the results being observed were due to CDD. These results suggest induction of autophagy in the MCF-7 cells by CDD.

## Discussion

This study characterized the cytotoxic potential of an important medicinal plant of the Arabian Peninsula, *C. droserifolia,* growing in Oman. It revealed that the dichloromethane fraction of the methanol extract, CDD (Fig. 1), had effective cytotoxic activity against human cancer cells (Table 1 & Fig. 1). CDD efficiently killed not only human breast but also cervical cancer cells in a dose- and time-dependent manner (Fig. 2). Investigation of the mechanism of action of CDD revealed that it had the ability to induce caspase 7, 8, and 9 enzymatic activity (Fig. 3) and activated cleavage of caspase 7 & PARP proteins (Fig. 4). Finally, FACS analysis of cells treated with CDD for Annexin V/PI staining confirmed that apoptosis could be induced by CDD which was reversible by the classical apoptosis inhibitor, Z-VAD-FMK (Figs. 6 & 7) in the hormonally-responsive MCF-7 breast cancer cell line.

These results support the observations made by Ezzat and colleagues on the *C. droserifolia* species from Egypt in two human cancer cell lines: MCF-7 and the colon carcinoma cell line, HCT116 [43]. Their tests revealed that both the aqueous and ethanolic extracts of the aerial parts of *C. droserifolia* had cytotoxic potential in these cell types. The aqueous extract was further fractionated using four organic solvents amongst which the chloroform, ethyl acetate, and *n*-hexane fractions were observed to have the most active cytotoxic potential, while the *n*-butanol fraction had weaker cytotoxic activity. Furthermore, nine compounds (six terpenoids and two flavanol glycosides) were isolated from the chloroform and ethyl acetate fractions of which two terpenoids and both flavonols had cytotoxic potential against the two cell lines, similar to those observed with the anticancer drug, Doxorubicin [43]. We only tested fractions of the methanolic extract for its cytotoxic potential. Furthermore, we have previously reported the isolation of two known aromatic derivatives, veratrol and 2-methoxy-4-methylacetophenone, and one novel alkaloid (5-hydroxy-2-methoxy-1-methy-1*H*-indole-3-carbaldehyde) from the dichloromethane fraction [30]; however, their anticancer cytotoxic potential remains to be tested owing to the insufficient availability of enough pure compounds.

Other than cytotoxicity for cancer cells, CDD also was able to kill normal human breast cells (Fig. 2). The ability of CDD to induce killing of normal cells is not surprising given the fact that we tested a crude organic fraction representing a mixture of compounds, and not a pure compound. Thus, it is possible that once the active biomolecules representing the cytotoxic principals have been isolated and separated from each other, we should be able to identify molecules that are able to induce death of only cancer and not normal cells. Even if that is not possible, our identification of crude fractions with anticancer potential, especially for breast cancer cells, is significant since it has been hard to identify natural compounds that have cytotoxicity for breast cancer cells in large screenings of natural compounds [64, 65].

Interestingly, we found that dichloromethane was the best solvent at extracting the cytotoxic potential from the methanolic extract. None of the other solvents could purify molecules with any significant cytotoxic activity. This observation agrees with our studies with other plant extracts where dichloromethane was the best at isolating the anticancer potential of the plant [52, 53]. These observations suggest that the dichloromethane solvent is one of the better organic solvents at extracting anticancer bioactive molecules from medicinal plants with cytotoxic effects against human cancer cells**.**

Finally, we also studied how treatment with a natural plant product like CDD can affect autophagy, an important cellular catabolic pathway responsible for maintaining cell health by engulfing damaged proteins and organelles, thereby recycling nutrients needed for cell survival [59]. We observed that CDD was efficient at inducing autophagy, as assessed by the induction and oligomerization of the autophagy receptor protein SQSTM1/p62 as well as activation of the cytosolic LC3-I form followed by its recruitment to the membrane-bound LC3-II (Fig. 8). We have observed similar effects of two other natural plant products on autophagy when apoptosis was induced by their organic fractions, although the precise pattern of induction of SQSTM1/p62 and LC3 was different with each plant product [52, 53]. In fact, this pattern differed even between the organic fraction from leaves (AOD (L)) versus the stems (AOB (S)) from the same plant, *Acridocarpus orientalis*. For example, AOD (L) had a lower potential of inducing apoptosis and was more efficient at inducing the dimer form of SQSTM1/p62 than AOB (S), while AOB (S) was more effective at inducing apoptosis and was also more efficient at inducing both forms of LC3 than AOD (L) [53]. These observations suggest that induction of apoptosis can have direct effects on autophagy, depending upon the nature and type of stress being imposed by the constituents of the medicinal plant product.

The role of autophagy in cancer cell death and survival is quite complex, especially in the presence of apoptosis [66, 67]. Autophagy has been shown to cause cell death along with apoptosis or ensure cell survival by inhibiting apoptosis [68]. Interestingly, it may even be a requirement for activating apoptosis in cells which are faced with stresses that can activate autophagy itself, such as oxidative stress or nutrient depravation [69, 70]. Thus, although we observed activation of autophagy in the case of CDD as well as other plant organic fractions [52, 53], it is not clear whether it was a requirement or a consequence of apoptotic cell death, manifesting as a cell survival response. For example, it has been shown that oligomerization of SQATM1/p62 by oxidative stress is suggestive of induction of autophagy as a pro-survival mechanism rather than a cell death process [69, 70]. Alternatively, it could also be an independent response to compounds in CDD or the other plant extracts. Thus, tweaking this out will require further careful experimentation with inhibitors of both apoptotic and autophagic pathways, followed by cellular and biochemical assays to determine the role being played by CDD or other natural compounds in the induction of autophagy.

## Conclusions

This study reveals that *C. droserifolia* has strong cytotoxic potential against human cancer cells. It further shows that the bioactive molecules in this plant exert their effect by inducing caspase-dependent apoptosis that could essentially be reversed by Z-VAD-FMK. Thus, *C. droserifolia* is a valuable medicinal plant in the fight against cancer which can also serve as a biological source of novel natural anticancer agents.

## Data Availability

The datasets used and/or analyzed during the current study are available from the corresponding author on reasonable request.

## Abbreviations

AOB (S): *Acridocarpus orientalis n*-butane stem fraction
AOD (L): *Acridocarpus orientalis* dichloromethane leaf fraction
CDB: *C. droserifolia n*-butanol fraction
CDD: *C. droserifolia* dichloromethane fraction
CDEA: *C. droserifolia* ethyl acetate fraction
CDH: *C.droserifolia n*-hexane fraction
DMEM: Dulbecco’s Modified Eagles Medium
DMSO: Dimethyl sulfoxide
EGF: Epidermal growth factor
FBS: Fetal bovine serum
FCS: Fetal calf serum
LC3: Microtubule-associated protein 1 light chain 3
MTT: [3-(4, 5-dimethylthiazol-2-yl)-2–5-diphenyltetrazolium bromide]
PARP: Poly (ADP-ribose) polymerase
PBS: phosphate-buffered saline
PBST: PBS and 0.1% Tween-20
PMSF: Phenylmethyl sulfonyl fluoride
RIPA: Radioimmunoprecipitation assay buffer
SDS: Sodium dodecyl sulfate
